# MCL1 participates in leptin-promoted mitochondrial fusion and contributes to drug resistance in gallbladder cancer

**DOI:** 10.1172/jci.insight.135438

**Published:** 2021-08-09

**Authors:** Wei-Jan Wang, Hong-Yue Lai, Fei Zhang, Wan-Jou Shen, Pei-Yu Chu, Hsin-Yin Liang, Ying-Bin Liu, Ju-Ming Wang

**Affiliations:** 1 Department of Biological Science and Technology and Research Center for Cancer Biology, China Medical University, Taichung, Taiwan.; 2 Department of Biotechnology and Bioindustry Sciences, College of Bioscience and Biotechnology, National Cheng Kung University, Tainan, Taiwan.; 3 Department of Medical Research, Chi Mei Medical Center, Tainan, Taiwan.; 4 Department of General Surgery, Xinhua Hospital Affiliated to Shanghai Jiao Tong University School of Medicine, Shanghai, China.; 5 Shanghai Research Center of Biliary Tract Disease, Shanghai, China.; 6 Graduate Institute of Biomedical Sciences, College of Medicine, China Medical University, Taichung, Taiwan.; 7 International Center for Wound Repair and Regeneration, National Cheng Kung University, Tainan, Taiwan.; 8 Graduate Institute of Medical Sciences, College of Medical Science and Technology, Taipei Medical University, Taipei, Taiwan.; 9 Graduate Institute of Medicine, College of Medicine, Kaohsiung Medical University, Kaohsiung, Taiwan.

**Keywords:** Endocrinology, Hepatology, Apoptosis survival pathways, Leptin, Mitochondria

## Abstract

Obesity is a risk factor for gallbladder cancer (GBC) development, and it correlates with shorter overall survival. Leptin, derived from adipocytes, has been suggested to contribute to the growth of cancer cells; however, the detailed mechanism of leptin in GBC drug resistance remains uninvestigated. In this study, our finding that patients with GBC with a higher BMI were associated with increased GBC risks, including shortened survival, is clinically relevant. Moreover, obese NOD/SCID mice exhibited a higher circulating concentration of leptin, which is associated with GBC growth and attenuated gemcitabine efficacy. We further revealed that leptin can inhibit gemcitabine-induced GBC cell death through myeloid cell leukemia 1 (MCL1) activation. The transcription factor C/EBP δ (CEBPD) is responsive to activated STAT3 (pSTAT3) and contributes to *MCL1* transcriptional activation upon leptin treatment. In addition, MCL1 mediates leptin-induced mitochondrial fusion and is associated with GBC cell survival. The findings in this study suggest the involvement of the pSTAT3/CEBPD/MCL1 axis in leptin-induced mitochondrial fusion and survival and provide a potentially new therapeutic target to improve the efficacy of gemcitabine in patients with GBC.

## Introduction

Obesity leads to cellular stress within adipocytes owing to surplus lipid accumulation, with a consequent release of adipokines and inflammatory cytokines from adipocytes and infiltrating immune cells, respectively. However, the key processes linking obesity and cancer remain unclear. Adipokine leptin has been suggested to be involved in the promotion of cell proliferation, metastasis, antiapoptosis, and angiogenesis (1, 2). The functions of leptin are mediated through the transmembrane leptin receptor (OBR) located in the hypothalamic nucleus by activation of the Janus-activated kinase/STAT3 (JAK/STAT3) and MAPK pathways (3). Gallbladder cancer (GBC) is an obesity-linked cancer that is associated with gallstones and chronic gallbladder inflammation. However, whether GBC cells receive leptin signals and the underlying mechanism of how leptin mediates antiapoptosis of GBC cells remain elusive.

Gemcitabine (Gemzar), as a DNA-damaging drug, is the most commonly used drug for GBC. However, it has been demonstrated that treatment with gemcitabine only reaches a 36% response rate (4). Meanwhile, obese patients with cancer have poorer outcomes than their leaner counterparts (5). Therefore, the precise mechanism linking obesity and poor therapeutic efficacy in GBC needs to be dissected. In addition, ATP synthesis in most tumor cells relies on glycolysis rather than oxidative phosphorylation, a phenomenon known as the Warburg effect. Interestingly, this hypothesis was initially attributed to mitochondrial dysfunction but is now being reevaluated. In contrast to Warburg’s first observation, maintaining functional mitochondria appears to be key for cancer cell survival and proliferation (6).

C/EBP δ (CEBPD) is one of the C/EBP family members that serves as a transcription factor and can be upregulated by a variety of extracellular stimuli, such as IL-6, IL-1β, LPS, IFN-α, IFN-γ, TNF-α (7), and modified LDL (8). CEBPD is also responsive to anticancer drugs, including vitamin D3 (9), hydroxymethyldibenzoylmethane (10), metformin (11), and bortezomib (12). Several studies have suggested that the p38^MAPK^/cAMP-responsive element–binding protein or JAK/STAT3 pathway plays important roles in the activation of *CEBPD* transcription (7). The inactivation of CEBPD has been observed in several types of cancers, including cervical cancer (10), hepatocellular carcinoma (13), breast cancer (14), prostate cancer (9), and leukemia (15). In addition to acting as a tumor suppressor, several recent reports have suggested that CEBPD plays an oncogenic role in certain conditions (16, 17). In bladder cancer, the attenuation of CEBPD can sensitize cisplatin-induced cell death in cisplatin-resistant bladder cancer cells (18).

Mitochondria, which are necessary for the production of energy by oxidative phosphorylation, are dynamic organelles that continually undergo fusion and fission. Mitochondrial fusion is driven by mitofusin 1 and 2 (MFN1/2) and optic atrophy 1 (OPA1). In addition, mitochondrial fission is driven by dynamin-related protein 1 (DRP1) and fission 1 (FIS1) (19). Myeloid cell leukemia 1 (MCL1), an antiapoptotic BCL-2 family member, is overexpressed in many types of human tumors, including leukemia (20), breast cancer (21), prostate cancer (22), and ovarian cancer (23); and its expression correlates with disease grade and survival-predicting response to anticancer therapies (24, 25). Increased recruitment of DRP1 to mitochondrial fission sites during apoptosis has been demonstrated (26). It was found that leptin stimulates fatty acid oxidation, glucose uptake, and ROS production in muscle, endothelial cells, and adipocytes (27–29), thus activating mitochondrial function (30). However, whether leptin promotes GBC survival by activating mitochondrial function remains unknown. Therefore, how MCL1 links mitochondrial fusion-fission dynamics and apoptotic cell death deserves to be dissected and could represent a new therapeutic target for the treatment of GBC.

## Results

### Obesity is associated with GBC progression.

**Figure 1:**
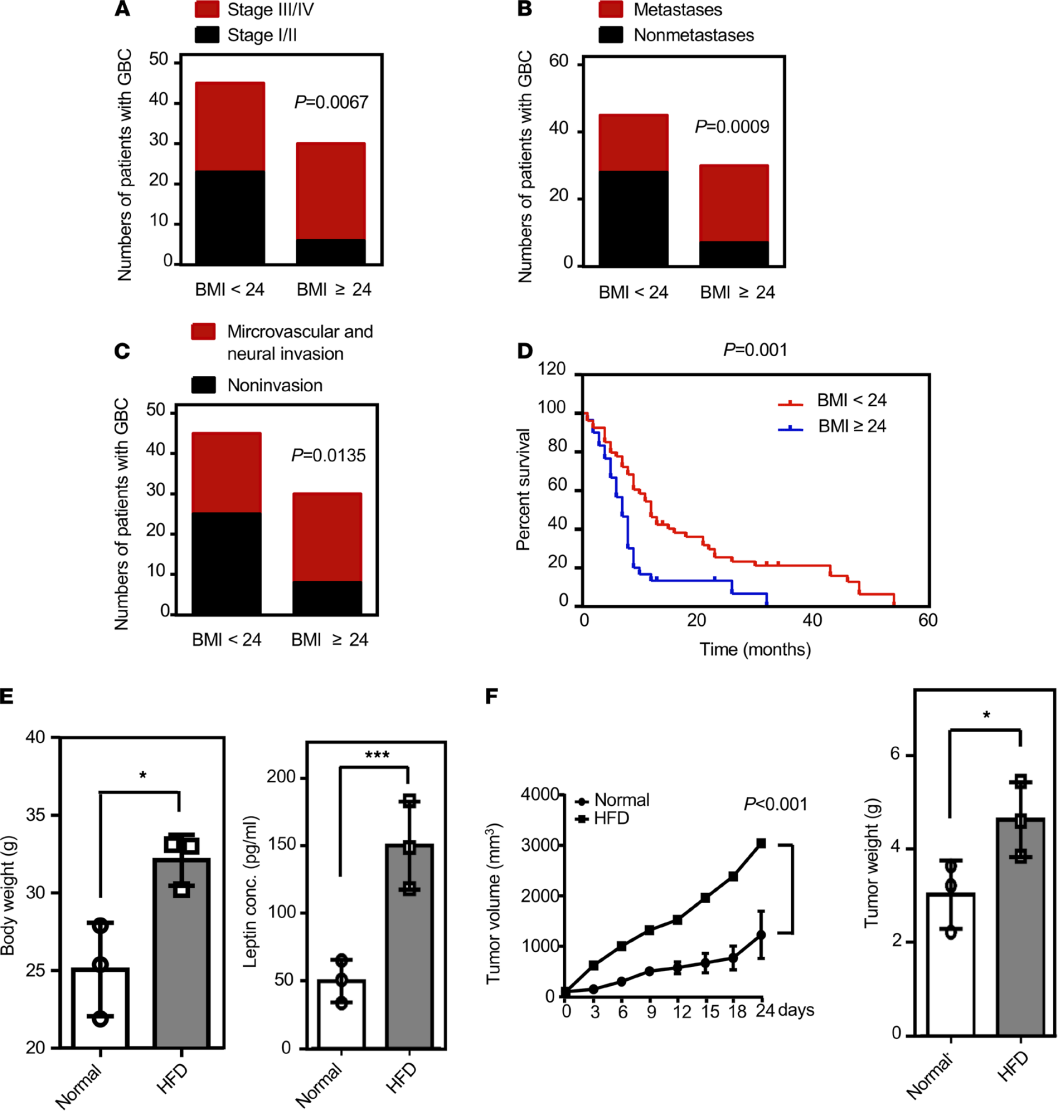
Evaluation of the significance of obesity in gallbladder cancer. (**A–C**) Numbers of patients at different clinical stages for those with BMI ≥24 (*n* = 30) and BMI <24 (*n* = 45). Numbers of metastases and nonmetastases for patients with BMI ≥24 and BMI <24. Numbers of instances of microvascular and nonmicrovascular invasion for patients with BMI ≥24 and BMI <24 (χ^2^ test). (**D**) Kaplan-Meier plots of the overall survival of patients with GBC based on BMI <24 (*n* = 45) or BMI ≥24 (*n* = 30) (Cox proportional regression analysis). (**E**) Four-week-old male NOD/SCID mice were fed with normal diet or high-fat diet for 12 weeks and then sacrificed at the age of 16 weeks. Body weights of normal diet– and high-fat diet–fed mice (*n* = 4; *P < 0.05) were recorded, and circulating leptin levels were measured by ELISA (*n* = 4; ***P < 0.0001, 2-tailed Student’s *t* test). (**F**) After being fed a normal or a high-fat diet for 12 weeks, mice were injected with GBC cells RCB-1130 (4 × 10^6^/mice). Tumors were allowed to grow for 14 days, and tumor volume was measured twice per week for 4 weeks using a caliper. Tumor volume was calculated according to the formula (length × width^2^)/2. The mice were sacrificed at the end of the experiment, and the tumors were removed and weighed (*n* = 4; *P < 0.05, 2-tailed Student’s *t* test).

To examine the effect of obesity on GBC progression, tumor tissue specimens and patient serum were obtained from 75 patients with GBC. We found that patients with gallbladder carcinoma with a BMI higher than or equal to 24 had higher clinical stages (*P* = 0.0067; Figure 1A) and were more likely to develop lymph node metastasis (*P* = 0.0009; Figure 1B) and neurovascular invasion (*P* = 0.0135; Figure 1C). In addition, the Cox proportional regression analysis with adjusted confounding factors revealed that the prognosis for patients with a BMI of less than 24 was significantly better than that for patients with BMI higher than or equal to 24 (*P* = 0.001; Figure 1D). Furthermore, to dissect the link between obesity and GBC, 4-week-old male NOD/SCID mice were fed with normal diet or high-fat diet (HFD) for 12 weeks. At the age of 16 weeks, the HFD-fed group exhibited increased body weight and elevated serum leptin levels compared with those of the normal diet–fed group (Figure 1E). We found that the growth curves of the size and weight of RCB-1130 tumor xenografts were significantly increased in HFD-fed mice compared with those of in control mice (Figure 1F). The results suggested that obesity promotes the progression of GBC in obese patients and HFD-fed mice.

### Adipocyte-conditioned medium protects GBC cells from apoptosis induced by gemcitabine.

**Figure 2:**
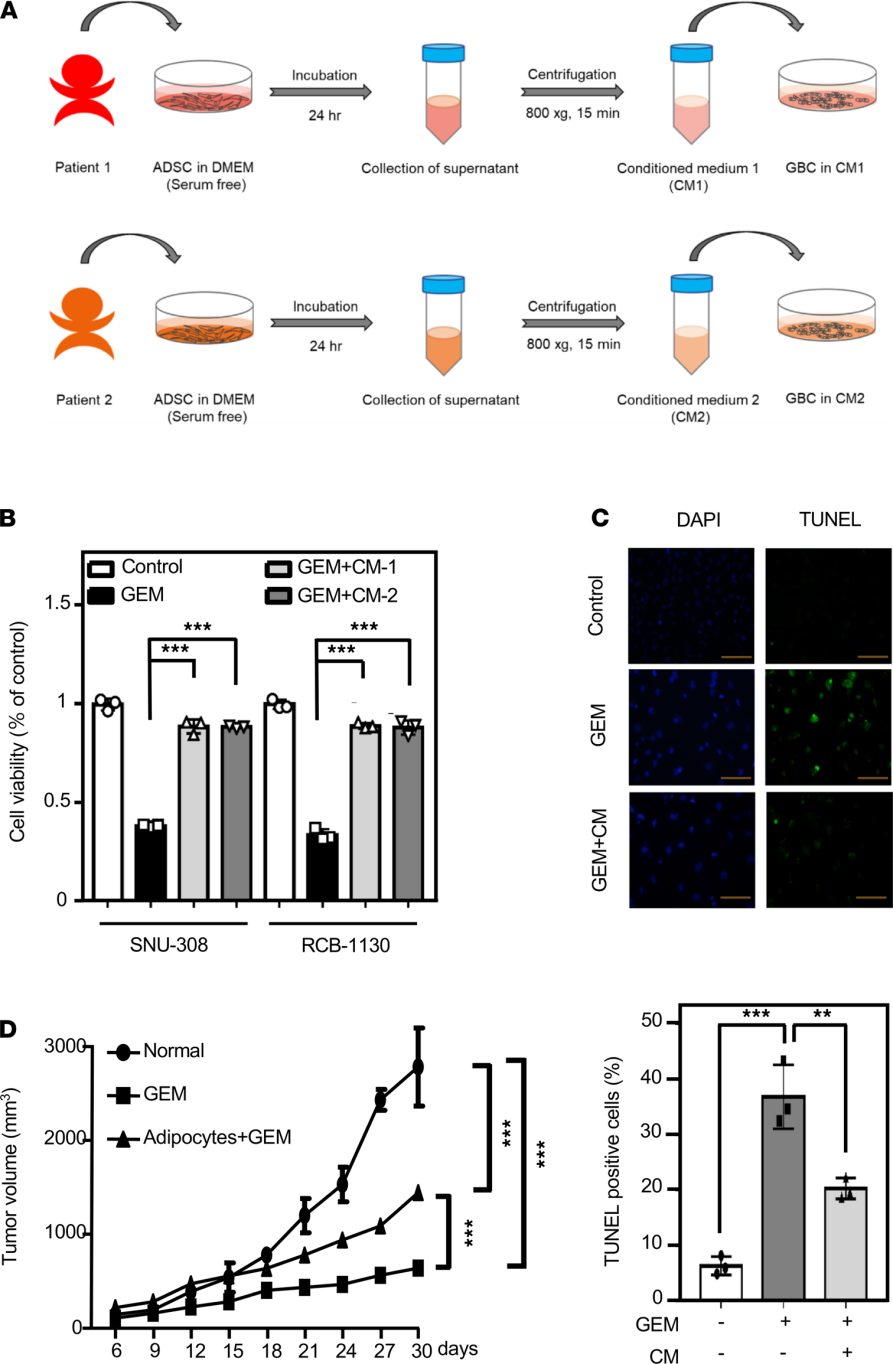
Adipocyte-conditioned medium benefits resistance of gallbladder cancer cells to gemcitabine-induced apoptosis. (**A**) Flow chart for producing conditioned medium from various adipocytes. (**B**) The viability of adipocyte-conditioned medium–treated GBC cells in response to gemcitabine (GEM). SNU-308 and RCB-1130 cells were treated with serum-free medium, adipocyte CM, and GEM for 24 hours in each sample. Cell viability was assessed by the MTT assay (*n* = 3; ***P < 0.001, 1-way ANOVA followed by Tukey’s multiple comparisons). (**C**) Inhibition of GEM-induced apoptosis by adipocyte CM treatment. SNU-308 and RCB-1130 cells were plated in 100 nM GEM or 100 nM GEM in combination with adipocyte CM and treated for 24 hours in microtiter plates. Cell apoptosis was analyzed by the TUNEL assay. Blue spots represent cell nuclei by DAPI staining, and green spots represent apoptotic bodies by TUNEL staining (*n* = 3; **P < 0.01, ***P < 0.001; 1-way ANOVA followed by Tukey’s multiple comparisons). Scale bar: 100 μm. (**D**) Four-week-old male NOD/SCID mice were fed a normal diet for 12 weeks. Then, mice were coinjected with gallbladder cancer RCB-1130 cells (4 × 10^6^/mice) and human primary adipocytes (1 × 10^6^/mice). Once the tumors attained a size of approximately 100 mm^3^, animals were randomized to receive gemcitabine treatment or were left untreated (control). Mice were treated twice per week by intraperitoneal injection of gemcitabine (400 mg/kg) diluted in 150 μL PBS, and tumor volume was measured for 4 weeks using a caliper. Tumor volume was calculated according to the formula (length × width^2^)/2 (*n* = 4; ***P < 0.001, 1-way ANOVA followed by Tukey’s multiple comparisons).

Adipocytes, the main cellular component of adipose tissue, are widely known to affect tumor behavior, including tumor growth and metastasis (31). To investigate whether adipocytes participate in the survival of GBC cells, we differentiated human adipose-derived stem cells (ADSCs) into adipocytes, and then the mature adipocyte gene marker *leptin* was used. Leptin mRNA levels were significantly increased in differentiated human primary adipocytes compared with levels in ADSCs (Supplemental Figure 1; supplemental material available online with this article; https://doi.org/10.1172/jci.insight.135438DS1). To further examine whether adipokines secreted from adipocytes effect the sensitivity of GBC cells to chemotherapeutic drugs, conditioned media (CM-1 or CM-2) from different donor-derived adipocytes were harvested for culture of SNU-308 and RCB-1130 cells that had been serum starved for 16 hours before addition of gemcitabine (Figure 2A). The results suggested that adipocyte CM significantly attenuated gemcitabine-induced GBC cell apoptosis (Figure 2, B and C). Moreover, a tumor xenograft assay further demonstrated that adipocytes could suppress gemcitabine-induced RCB-1130 tumor xenograft death (Figure 2D). These results suggested that adipocytes facilitate gemcitabine resistance of GBC cells.

### Leptin attenuates gemcitabine-induced GBC cell apoptosis.

**Figure 3:**
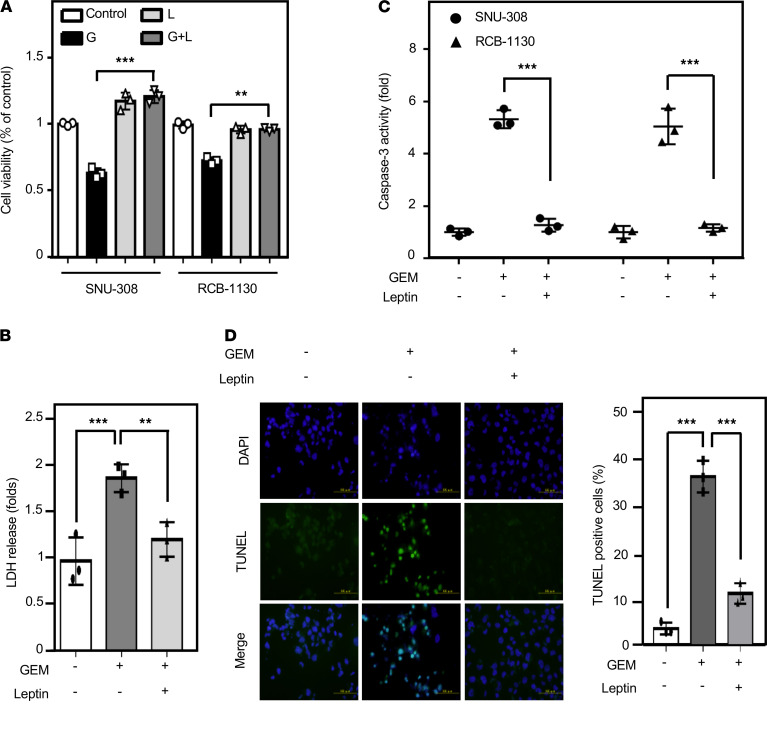
Leptin rescues gallbladder cancer cells from gemcitabine-induced cell apoptosis. (**A**) The viability of leptin–treated GBC cells in response to gemcitabine (GEM). SNU-308 and RCB-1130 cells were treated with GEM alone or in combination treatment with leptin. Cell viability was assessed by the MTT assay (*n* = 3; ***P < 0.001, **P < 0.01, 2-tailed Student’s *t* test). (**B**) RCB-1130 cells were starved for 24 hours and then treated with 100 nM gemcitabine or a combination of gemcitabine and leptin (100 ng/mL) for 24 hours. Cell toxicity was measured by the LDH assay (*n* = 3; **P < 0.01, ***P < 0.001), 1-way ANOVA followed by Tukey’s multiple comparisons). (**C**) Leptin attenuates GEM-induced caspase-3 activation. Caspase-3 activity was detected in SNU-308 and RCB-1130 cells (*n* = 3; ***P < 0.001, 2-tailed Student’s *t* test). (**D**) SNU-308 and RCB-1130 cells were plated in 100 nM GEM or 100 nM GEM in combination with leptin and treated for 24 hours in microtiter plates. Cell apoptosis was analyzed by the TUNEL assay. Blue spots represent cell nuclei by DAPI staining, and green spots represent apoptotic bodies by TUNEL staining (*n* = 3, 1-way ANOVA followed by Tukey’s multiple comparisons). Scale bar: 100 μm.

Leptin, the major adipocyte-derived adipokine, is a known biomarker of obesity due to the high positive correlation of its circulating levels with BMI. However, the details of leptin-mediated antiapoptosis remain uninvestigated in GBC cells. We first confirmed the expression of the OBR in both SNU-308 and RCB-1130 cells (Supplemental Figure 2) to ensure that GBC cells are capable of receiving leptin signals. Leptin has been suggested to be an oncogenic factor, and we further tested the effect of leptin on cell viability and cytotoxicity by MTT and lactate dehydrogenase s (LDH) assays, respectively. The results showed that leptin significantly promoted GBC cell survival upon gemcitabine treatment (Figure 3, A and B). Moreover, the effect of leptin on antiapoptosis was checked by the level of caspase-3 cleavage and the TUNEL assay. In addition to the CM from adipocytes, leptin attenuated gemcitabine-induced apoptosis in GBC cells (Figure 3, C and D). These results suggested that leptin contributes to gemcitabine resistance of GBC cells.

### STAT3 contributes to the survival effect of leptin through CEBPD activation.

**Figure 4:**
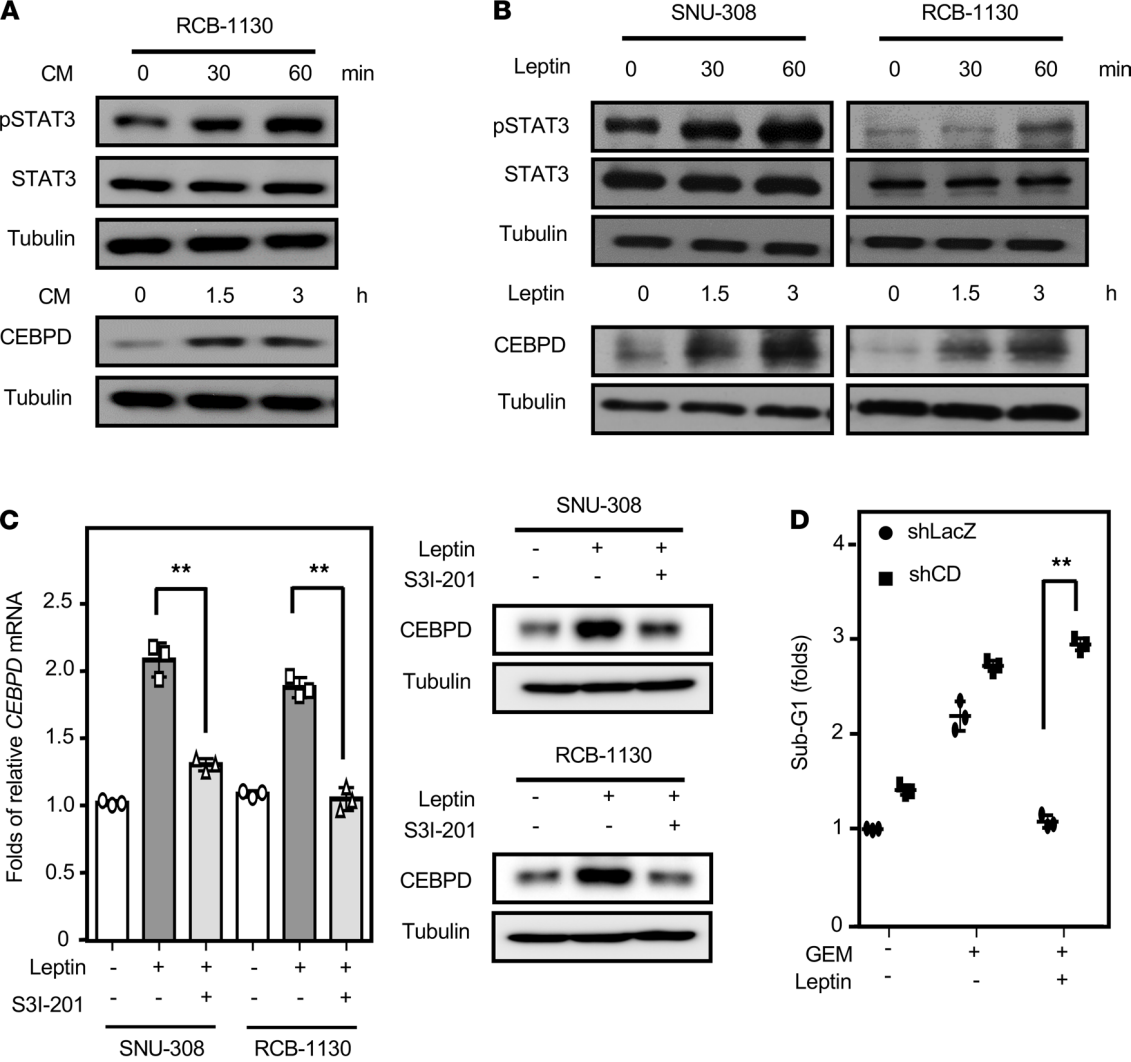
STAT3 mediates leptin-induced gallbladder cancer cell survival through CEBPD activation. (**A**) Human adipocyte CM induces pSTAT3/CEBPD expression. RCB-1130 cells were exposed to adipocyte CM, and lysates were harvested according to the indicated time courses (*n* = 3). (**B**) pSTAT3/CEBPD expression following leptin treatment in GBC cells. SNU-308 and RCB-1130 cells were treated with leptin, and lysates were harvested according to the indicated time courses. Antibodies recognizing pSTAT3 (pY705), STAT3, CEBPD, and α-tubulin were used in Western blot analysis (*n* = 3). (**C**) STAT3 inhibitor (S3I-201) attenuated leptin-induced CEBPD mRNA and protein expression in GBC cells. Western blot analysis (*n* = 3) was conducted with lysates and qPCR assays (*n* = 3; **P < 0.01, 2-tailed Student’s *t* test) were conducted with total RNA harvested from SNU-308 and RCB-1130 cells. (**D**) Attenuation of CEBPD in RCB-1130 cells sensitizes them to gemcitabine (GEM) upon leptin treatment. Cells were pretreated with lentiviruses containing shLacZ (LacZ) or shCEBPD (CDKD). After 48 hours of incubation, experimental cells were treated with or without leptin and GEM. Death of experimental cells was examined by PI staining (*n* = 3; **P < 0.01, 2-tailed Student’s *t* test).

The induction of CEBPD expression is associated with hepatic lipogenesis (32) and macrophage lipid accumulation (8), and Western blot analyses revealed that adipocyte CM and leptin can induce the expression of CEBPD in GBC cells (Figure 4, A and B). Previous studies have indicated that the STAT3 pathway is important for leptin signaling and CEBPD activation (33), but it had not been tested in GBC cells. Western blotting demonstrated that adipocyte CM and leptin could induce the phosphorylation of STAT3 in GBC cells (Figure 4, A and B). To test whether STAT3 activity mediates leptin-induced CEBPD expression, the STAT3 inhibitor S3I-201 was used. RT-PCR and Western blot analyses showed that S3I-201 could suppress leptin-induced CEBPD expression in SNU-308 and RCB-1130 cells (Figure 4C). Furthermore, to assess whether CEBPD contributes to the antiapoptotic effect of leptin, a loss-of-function assay using lentiviruses encoding shLacz or shCEBPD was conducted. The results showed that the knockdown of CEBPD restored gemcitabine-induced RCB-1130 cell apoptosis upon leptin treatment (Figure 4D). Taken together, these results suggested that STAT3 and its downstream target CEBPD mediate leptin-induced GBC cell survival.

### MCL1 is a target gene of CEBPD in response to leptin and is associated with GBC progression.

**Figure 5:**
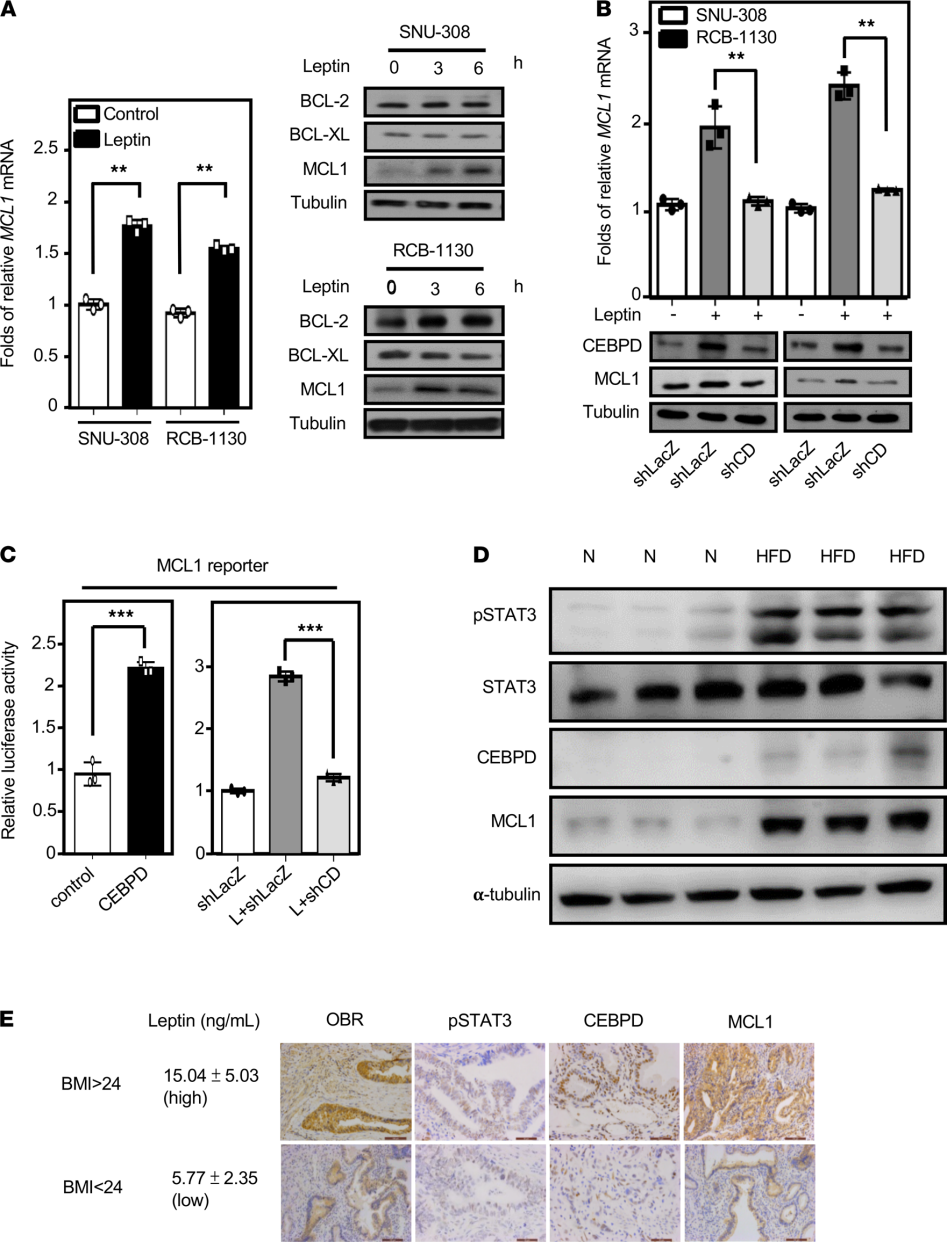
CEBPD activates *MCL1* gene transcription in response to leptin and is involved in gallbladder cancer progression. (**A**) Leptin induces MCL1 expression. Western blot analysis (*n* = 3) was conducted with lysates, and qPCR assays (*n* = 3; **P < 0.01, 2-tailed Student’s *t* test) were conducted with total RNA harvested from SNU-308 and RCB-1130 cells treated with leptin following various time courses. (**B**) Loss of CEBPD attenuates leptin-induced MCL1 expression. Cells were pretreated with lentiviruses containing shLacZ (LacZ) or shCEBPD (CDKD). After 48 hours of incubation, experimental cells were treated with leptin. qPCR assays (*n* = 3; **P < 0.01, 2-tailed Student’s t test) were conducted with total RNA, and Western blot analysis (*n* = 3) was conducted with lysates harvested from SNU-308 and RCB-1130 cells. (**C**) Loss of CEBPD attenuates leptin-induced MCL1 reporter activity. Graphs show cells transfected with expression vectors with or without CEBPD cDNA (CD and CTL, respectively) and cells that were pretreated with lentiviruses containing shLacZ (LacZ) or shCEBPD (CDKD). After 48 hours of incubation, experimental cells were treated with leptin. qPCR assays were conducted with total RNA harvested from SNU-308 and RCB-1130 cells (*n* = 3; ***P < 0.001, 2-tailed Student’s *t* test). (**D**) The pSTAT3/CEBPD/MCL1 axis is higher in RCB-1130 xenograft HFD-fed mice. After injecting gallbladder cancer RCB-1130 cells into normal and HFD-fed obese mice for 4 weeks, the xenograft mice were sacrificed to extract tumor xenografts. The protein expression was then examined by Western blot (*n* = 3). (**E**) Serum leptin levels and OBR, p-STAT3, CEBPD, and MCL1 expression were detected by ELISA and IHC (*n* = 75). Scale bar: 100 μm.

MCL1, an antiapoptotic BCL-2 family member that is essential for cell survival, is highly amplified in many types of cancer. IHC staining revealed that MCL1 expression was higher in RCB-1130 tumor xenografts in HFD-fed mice compared with those in normal diet–fed mice (Supplemental Figure 3A). We also found that leptin can specifically induce the expression of MCL1, but not BCL-2 and BCL-XL, in SNU-308 and RCB-1130 cells (Figure 5A). We further tested whether CEBPD regulates MCL1 expression. The results of the loss-of-function assay showed that the knockdown of CEBPD attenuated leptin-induced *MCL1* gene and protein expression (Figure 5B). Next, the results of the reporter assay showed that CEBPD could upregulate the activity of the *MCL1* reporter (Figure 5C). In contrast, the knockdown of CEBPD attenuated leptin-induced *MCL1* reporter activity (Figure 5C). These results suggested that CEBPD contributes to *MCL1* gene transcription in GBC cells upon leptin stimulation.

To corroborate the in vitro data, we used the tumor xenografts from HFD-fed mice to clarify the expression of pSTAT3, CEBPD, and MCL1. In keeping with previous results, the pSTAT3/CEBPD/MCL1 axis was upregulated in the HFD-fed tumor xenografts. However, IHC staining showed that the expression of leptin (an adipocyte marker) and α-SMA (a fibroblast marker) was marginally induced in the HFD-fed tumor xenografts (Figure 5D and Supplemental Figure 3B), suggesting that circulating leptin may play a more vital role for the development of HFD-fed tumor xenografts. Furthermore, to investigate the relationship among BMI and leptin levels and OBR, pSTAT3, CEBPD, and MCL1 expression, patient serum and tumor specimens were evaluated by ELISA and IHC, respectively. We found that serum leptin levels were significantly increased and that the abundance of OBR, pSTAT3, CEBPD, and MCL1 also correspondingly increased in patients with GBC with a BMI higher than or equal to 24 (Figure 5E and Table 1), but there was no statistical significance differences when comparing sex, age, and the pSTAT3/CEBPD/MCL1 axis (Supplemental Table 1). In addition, the expression levels of pSTAT3, CEBPD, and MCL1 were positively correlated (Table 2). To further investigate the correlations among pSTAT3, CEBPD, and MCL1 and the progression of GBC, the levels of pSTAT3, CEBPD, and MCL1 were examined in 75 patients with GBC. A clinicopathologic association study in patients with GBC demonstrated that high expression of pSTAT3, CEBPD, and MCL1 was significantly associated with advanced clinical stages, tumor grades, and lymph node metastasis (Supplemental Table 1). Taken together, these results indicated that high levels of serum leptin and pSTAT3/CEBPD/MCL1 axis activation were significantly associated with advanced clinical stages and tumor grades.

### Inhibition of the CEBPD/MCL1 axis strengthens gemcitabine-induced apoptosis.

**Figure 6:**
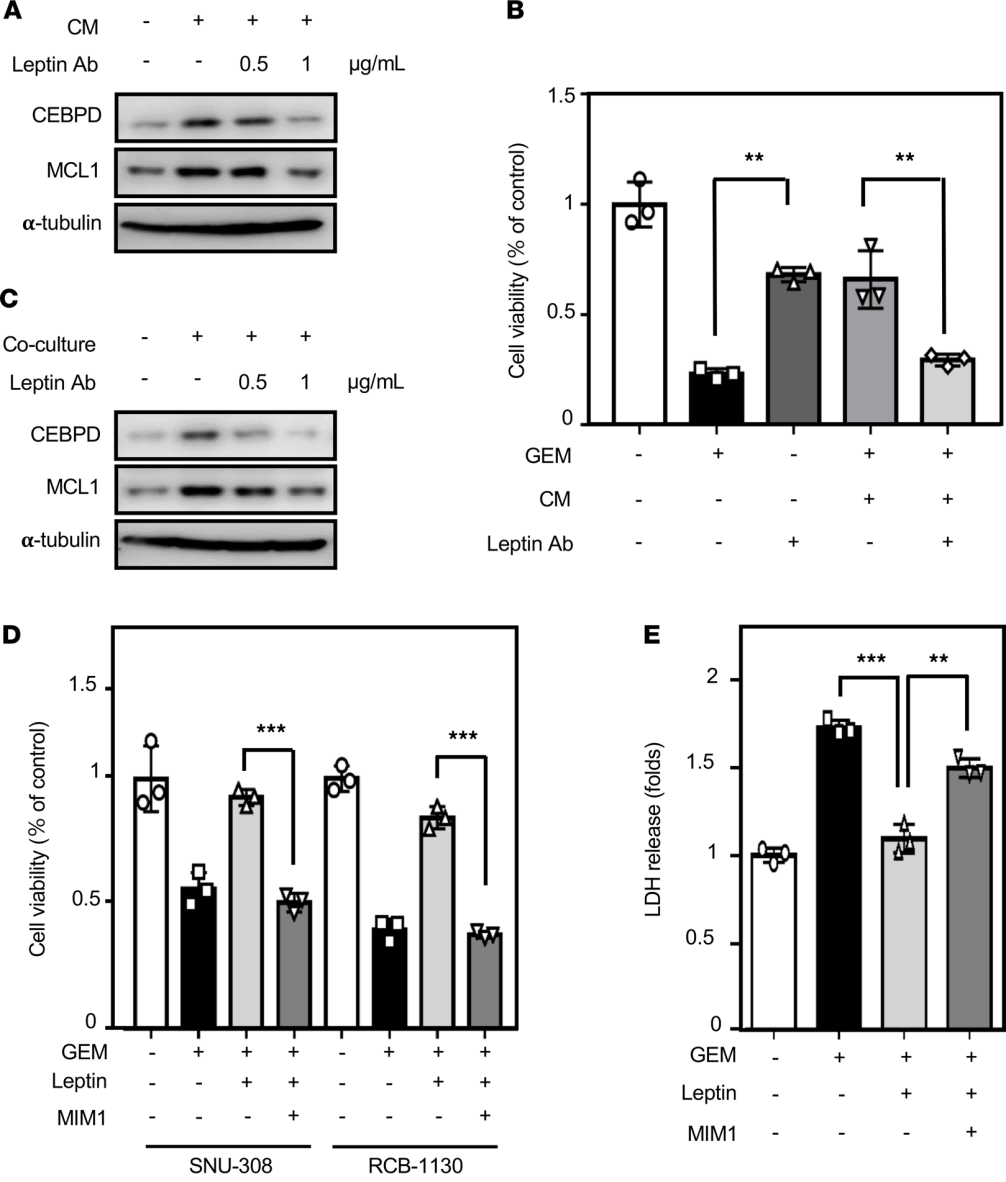
Inhibition of the CEBPD/MCL1 axis enhances gemcitabine-induced apoptosis. (**A**) Attenuation of leptin by neutralized antibody MAB398 inhibits CEBPD and MCL1 expression in RCB-1130 cells. RCB-1130 cells were treated with leptin and with or without MAB398, and lysates were harvested at the indicated concentrations. Antibodies recognizing CEBPD, MCL1, and α-tubulin were used in Western blot analysis (*n* = 3). (**B**) Neutralization of leptin resensitizes RCB-1130 cells to GEM. RCB-1130 cells were treated with GEM alone or with GEM in combination with leptin and with or without MAB398. Cell viability was assessed by MTT assay (*n* = 3; **P < 0.01, 1-way ANOVA followed by Tukey’s multiple comparisons). (**C**) Attenuation of leptin inhibits adipocyte-induced CEBPD and MCL1 expression in RCB-1130 cells. Transwell coculture system was used in this study. After 12 hours of coculture with adipocytes, the expression of CEBPD and MCL1 in RCB-1130 cells was examined by Western blot analysis (*n* = 3). (**D** and **E**) The MCL1 inhibitor MIM1 enhances gemcitabine (GEM) sensitivity in leptin-treated GBC cells. SNU-308 and RCB-1130 cells were treated with GEM alone or with GEM in combination with leptin and MIM1 for 24 hours. Cell viability was detected by the MTT assay (*n* = 3; ***P < 0.001, 2-tailed Student’s *t* test). Cell toxicity was measured by the LDH assay (*n* = 3; **P < 0.05, 1-way ANOVA followed by Tukey’s multiple comparisons).

A recent study showed that PPARγ activation can block activation of OBR and the JAK/STAT3 signaling pathway (34). To clarify the role of the CEBPD/MCL1 axis in leptin-induced survival of GBC cells, a PPARγ agonist, rosiglitazone, was applied to address this issue. Western blotting showed that rosiglitazone could suppress leptin-induced expression of CEBPD and MCL1 in SNU-308 and RCB-1130 cells (Supplemental Figure 4A). As analyzed by flow cytometry with PI staining, leptin significantly rescued SNU-308 and RCB-1130 cells from gemcitabine-induced cell apoptosis; moreover, rosiglitazone blocked the rescue of leptin (Supplemental Figure 4B). Furthermore, the leptin neutralized antibody MAB398 suppressed adipocyte CM-induced CEBPD and MCL1 expression (Figure 6A) and significantly attenuated adipocyte CM-rescued RCB-1130 cell survival (Figure 6B). Moreover, adipocyte coculture assay showed that MAB398 suppressed adipocyte-induced CEBPD and MCL1 expression in GBC cells (Figure 6C). To specifically assess the role of MCL1 in leptin-induced GBC cell survival, the MCL1 inhibitor MIM1 was used. We found that treatment with MIM1 significantly inhibited the leptin-induced survival effect on gemcitabine-treated SNU-308 and RCB-1130 cells (Figure 6D). LDH assays also demonstrated that MIM1 could enhance gemcitabine-induced apoptosis in leptin-treated RCB-1130 cells (Figure 6E). Collectively, these findings suggested that the CEBPD/MCL1 axis mediates the effect of leptin against gemcitabine-induced GBC cell apoptosis.

### Inhibition of MCL1 enhances mitochondrial fission and gemcitabine efficacy in GBC cells.

**Figure 7:**
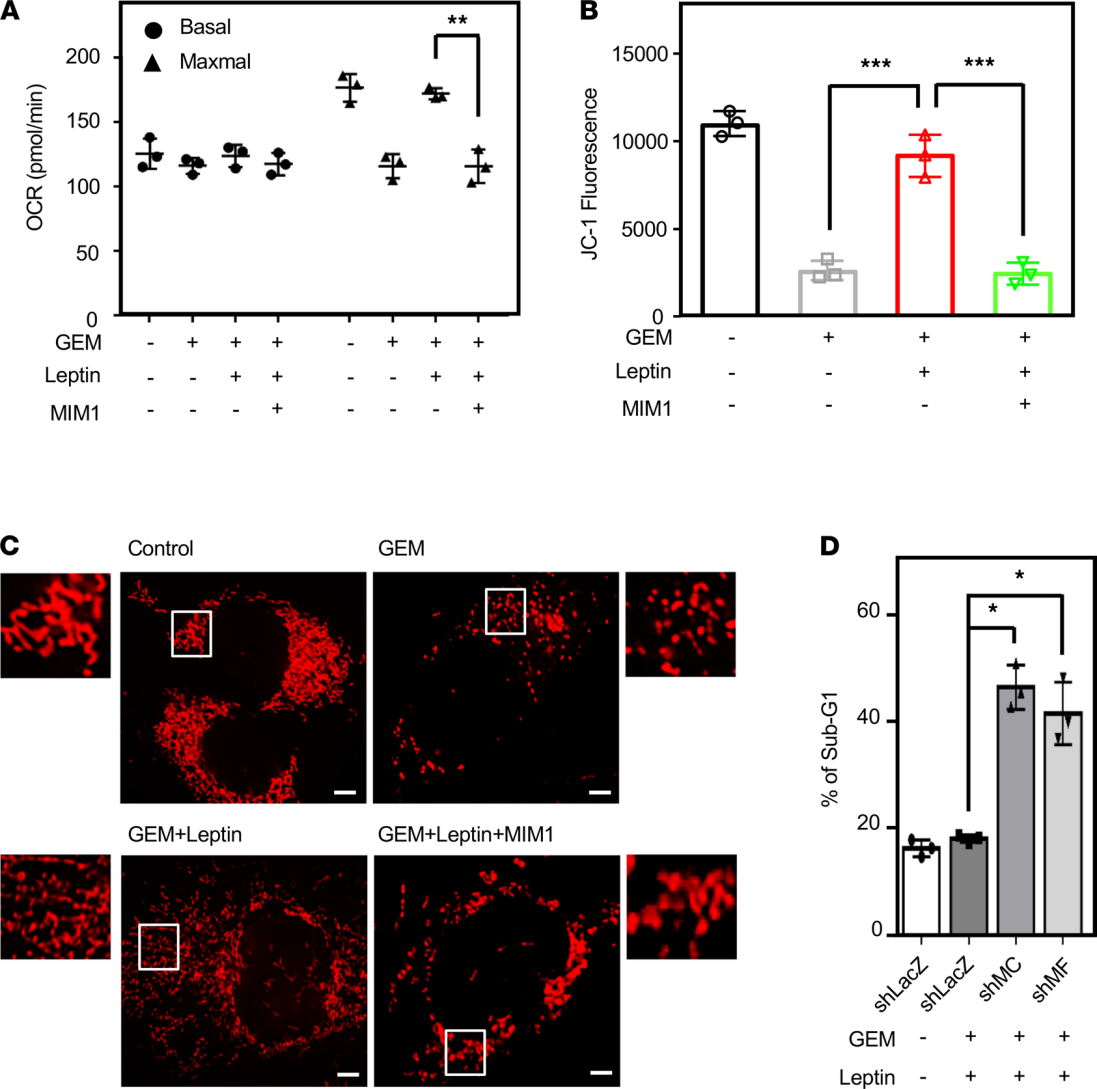
MCL1 promotes mitochondrial fusion and attenuates gemcitabine efficacy in gallbladder cancer cells. (**A**) MCL1 inhibitor MIM1 induces mitochondrial stress. Maximum oxygen consumption rate (OCR) was stimulated by FCCP addition (*n* = 3; **P < 0.01, 2-tailed Student’s *t* test). (**B**) MCL1 inhibitor decreases mitochondrial membrane potential in RCB-1130 cells. Quantification of JC-1 staining is used as an indicator of membrane potential (*n* = 3; ***P < 0.001, 1-way ANOVA followed by Tukey’s multiple comparisons). (**C**) The MCL1 inhibitor MIM1 promotes GEM-induced mitochondrial fission in leptin-treated GBC cells. SNU-308 and RCB-1130 cells were treated with GEM alone or GEM in combination with leptin and MIM1 for 24 hours. Confocal immunofluorescence of ectopic expression in RCB-1130 cells that were loaded with MitoTracker (*n* = 3). Scale bar: 10 μm. (**D**) Loss of MCL1 and MFN1 enhances GEM sensitivity upon leptin treatment. Cells were pretreated with lentiviruses containing shLacZ (LacZ), shMCL1 (shMC), or shMFN1 (shMF). After 48 hours of incubation, experimental cells were treated with leptin in combination with GEM. Death of experimental cells was examined by PI staining (*n* = 3; *P < 0.05, 1-way ANOVA followed by Tukey’s multiple comparisons).

Recently, mitochondrial dynamics have played a critical role in cancer cell death. To elucidate mitochondrial function upon leptin treatment, a seahorse assay was performed to detect the oxygen consumption rate (OCR), a mitochondrial stress index. The results showed that leptin significantly raised maximal OCR in RCB-1130 cells upon gemcitabine treatment. Meanwhile, MIM1 inhibited the leptin-raised maximal OCR (Figure 7A). Moreover, we further performed a mitochondrial membrane potential (MMP) assay to validate the function of mitochondria. In consistent with OCR results, MIM1 also inhibited leptin-increased MMP (Figure 7B). We further assessed the effect of MIM1 on mitochondrial dynamics. MitoTracker staining showed that leptin could inhibit gemcitabine-induced mitochondrial fission; however, MIM1 increased mitochondrial fission in RCB-1130 cells cotreated with gemcitabine and leptin (Figure 7C). Moreover, the knockdown of MCL1 or the mitochondrial fusion marker MFN1 increased gemcitabine sensitivity in leptin-treated RCB-1130 cells (Figure 7D). The results suggested that MCL1 plays a critical role in leptin-induced GBC cell survival via the promotion of mitochondrial fusion and mitochondrial function. These results also indicated that targeting MCL1 can improve gemcitabine sensitivity in GBC cells.

## Discussion

**Figure 8:**
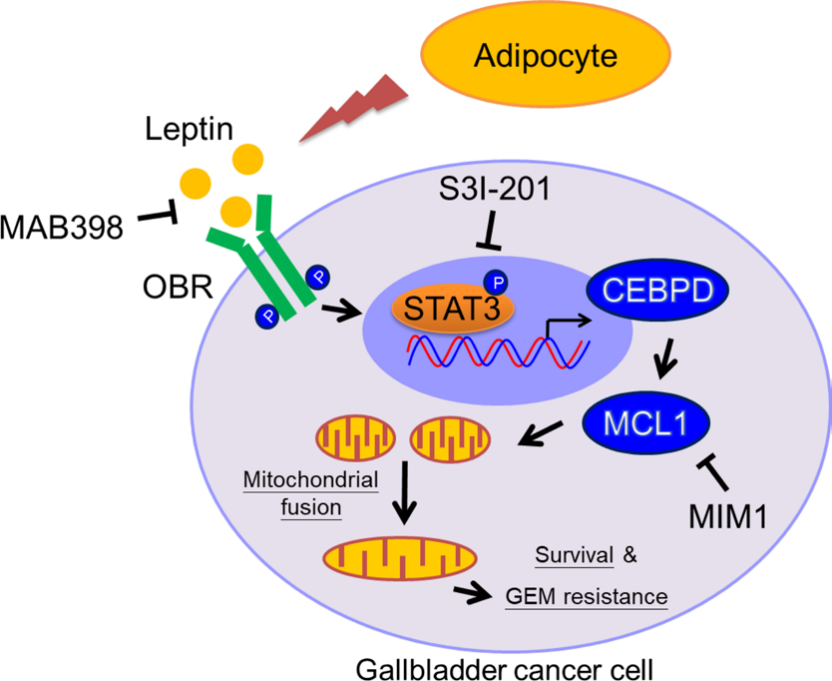
Involvement of the pSTAT3/CEBPD/MCL1 axis in leptin-induced mitochondrial fusion and survival. In response to leptin, the STAT3 pathway contributes to CEBPD/MCL1 activation. The activation of MCL1 protects GBC cells from gemcitabine-induced apoptosis through increased mitochondrial fusion and sustained mitochondrial function. The inhibitors of leptin, STAT3, or MCL1 can attenuate mitochondrial fusion and enhance sensitization of GBC cells to gemcitabine.

GBC is an obesity-linked disease that is associated with poor drug efficiency. Although the scientific principles that link obesity and cancer are broad, most researchers have largely focused on hormone-sensitive cancers, in particular breast and prostate cancer, or on specific adipokines and growth factors, including leptin, adiponectin, insulin, and insulin-like growth factor 1 (IGF-1). However, the details, including the molecular mechanisms related to cell survival and drug resistance, are still largely unknown. In this study, patients with GBC with a higher BMI (BMI ≥24 kg/m^2^) are associated with increased GBC risks, and the HFD-fed tumor xenograft model supports the obesity contribution in GBC cell growth. Moreover, we demonstrated that leptin can inhibit gemcitabine-induced GBC cell death via MCL1 activation. In response to leptin, the pSTAT3 pathway contributes to CEBPD/MCL1 activation, which consequently promotes gemcitabine-treated GBC cell survival via an increase in mitochondrial fusion. Furthermore, the expression of pSTAT3, CEBPD, and MCL1 is associated with GBC progression, as determined by the assessment of clinical specimens. This study underscores how MCL1 functions at the junction of mitochondrial fusion and survival and provides a therapeutic target to improve the efficacy of GBC treatment (Figure 8).

Chemoresistance in several types of cancer has been linked to the activation of STAT3 (33). STAT3 has been suggested to confer enhanced survival abilities following genotoxic treatments (35). Inhibition of the STAT3 pathway has been demonstrated to result in growth arrest, apoptosis, and chemosensitivity in several models of human malignancies (36). Several STAT3 inhibitors have also entered clinical trials for obesity-linked cancers, including pancreatic cancer (phase III), metastatic colorectal cancer (phase II), ovarian cancer (phase II), hepatocellular carcinoma (phase I), and breast cancer (phase I) (37). Here, this study suggests that the STAT3 inhibitor S3I-201 could also be beneficial for obese patients with GBC. MCL1 is one of the most highly amplified genes in a variety of cancers. Furthermore, its expression is often associated with chemotherapy drug resistance (38). This implies that MCL1 can be a therapeutic target to improve the efficacy of cancer treatment. Here, regarding the result that the MCL1 inhibitor MIM1 enhances the sensitivity of GBC cells to gemcitabine, we propose that MCL1 inhibition should be an attractive target for the improvement of the efficacy of gemcitabine. Many small-molecular inhibitors could be tested with the goal of MCL1 inhibition in cancer cells. For example, UMI-77 can antagonize MCL1 function by blocking the heterodimerization of MCL1/BAX and MCL1/BAK and exhibits tumor inhibitory activity in a triple-negative breast cancer cell xenograft mouse model (39). Furthermore, Obatoclax (GX15-070) is a novel BH3 mimetic that has been shown to interfere with the direct interaction between MCL1 and BAK and overcomes MCL1-mediated resistance to apoptosis (40). However, most of the MCL1 inhibitors are still at the preclinical or early clinical development stages. Further clinical trials are needed to evaluate the safety and efficacy of these compounds.

Mitochondria play a central role in apoptosis that is regulated by BCL-2 family members. Mitochondrial fission is an early step during apoptosis, occurring before caspase activation. A previous study showed that proapoptotic BAX and BAK promote fragmentation of the mitochondrial network, possibly by activating the mitochondrial fission machinery (41). The antiapoptotic protein MCL1 can block the progression of apoptosis by neutralizing BAX- or BAK-activated apoptotic activity. However, the link between MCL1 and mitochondrial dynamics in gemcitabine resistance of GBC cells is still unknown. In this study, we demonstrated that the MCL1 inhibitor MIM1 can enhance mitochondrial fission and promote gemcitabine sensitivity in leptin-treated GBC cells. These results imply that MCL1 can sustain mitochondrial function to maintain GBC cell survival. To date, the evolutionary hypothesis of mitochondria is that a symbiotic bacterium resided inside a protoeukaryotic cell and exchanged safely for energy. However, this symbiosis might create a survival advantage, such as the one described here. Therefore, whether and how MCL1-promoted mitochondrial fusion is involved in drug resistance in obese patients with cancer deserves further investigation, and this process can serve as a therapeutic target for the treatment of cancers.

Dysregulation of the leptin/leptin receptor has been suggested to participate in the development of a large variety of malignancies, including breast cancer, pancreatic cancer, thyroid cancer, endometrial cancer, and gastrointestinal cancer, predominantly through the JAK/STAT pathway (42, 43). Inactivation of CEBPD has been suggested to benefit several types of cancer development (7), implying that it has a tumor-suppressive role. However, several studies indicated that the activation of CEBPD is associated with drug resistance and stemness (33, 44). In addition, mitochondria are increasingly recognized as key drivers in the origin and development of cancer stem cell functional traits (45). Disruption of mitochondrial fusion is associated with induction of apoptosis (46), and mitochondrial fusion drives stem cell formation (47) in breast cancer. In response to leptin, we demonstrated the activation of the pSTAT3/CEBPD/MCL1 axis in GBC. Meanwhile, the axis activation is associated with mitochondrial fusion. However, whether the activation of the pSTAT3/CEBPD/MCL1 axis plays a common and vital role in the development of leptin-associated cancers as well as the idea of combined targeting of STAT3 and MCL1 to block mitochondrial fusion and stemness activity for GBC therapy deserve to be investigated in the future.

## Methods

### Patients and clinicopathological data.

Tumor tissue specimens and patient sera were obtained from 75 patients with GBC who had undergone radical cholecystectomy, without any prior radiotherapy or chemotherapy, between 2008 and 2015, at the Department of General Surgery, Xinhua Hospital, School of Medicine, Shanghai Jiao Tong University. The 75 Chinese patients with GBC included 21 men and 54 women. Survival information for patients was collected through phone calls. All diagnoses of GBC and lymph node metastasis were confirmed by histopathological examination, and all tissue samples were fixed in 4% formalin immediately after removal and embedded in paraffin for immunohistochemical staining.

### Immunohistochemical analysis of GBC tissues.

Following deparaffinization and quenching of endogenous peroxidase, sections were incubated with 1% bovine serum albumin in PBS. Subsequently, the slides were treated with rabbit primary antibodies against OBR, p-STAT3, CEBPD, and MCL1 followed by incubation with goat anti-rabbit IgG antibodies. The slides were counterstained with ChemMate Hematoxylin (Dako Cytomation) and mounted and observed under a BX51 microscope (Olympus). Sections were semiquantitatively scored twice for the extent of immunoreaction as follows. For the first score, positive cells in less than 5% of the total indicated a score of 0; positive cells in 5%–25% indicated a score of 1; positive cells in 25%–50% indicated a score of 2; and positive cells in greater than 50% of the total indicated a score of 3. For the second score, no coloration was scored as 0; pale yellow was scored as 1; yellow was scored as 2; and brown was scored as 3. The 2 scores were then multiplied; the resulting combined scores were categorized as follows: a score of 0 was negative (–); scores of 1–3 indicated weak immunoreactivity (+); scores of 4–6 indicated moderate immunoreactivity (++); and scores of 7–9 indicated strong immunoreactivity (+++).

### Xenograft model.

Four-week-old male NOD/SCID mice were purchased from NCKU Laboratory Animal Center. The mice were fed with normal diet or HFD (60% kcal from fat) for 12 weeks. RCB-1130 cells (4 × 10^6^ cells in 100 μl PBS) were injected subcutaneously into mice, and tumor formation was observed twice per week for 4 weeks. For treatment studies, 4-week-old male NOD/SCID mice were fed normal diet for 12 weeks, and then mice were coinjected with GBC RCB-1130 cells (4 × 10^6^/mice) and human primary adipocytes (1 × 10^6^/mice). Once the tumors attained a size of approximately 100 mm^3^, animals were randomized to receive gemcitabine (400 mg/kg) diluted in 0.1 ml PBS or PBS only (normal control) (*n* = 3 per group) twice per week by intraperitoneal injections. Tumor formation was observed twice per week for 4 weeks. Tumor volume was measured using caliper and calculated according to formula (length × width^2^)/2.

### Mouse leptin ELISA assay.

The blood samples were collected by cardiac puncture from normal diet– or HFD-fed mice. The blood samples were allowed to clot undisturbed for 30 minutes at room temperature. The sera were separated from the blood samples by centrifugation at 239 g for 15 minutes. The serum leptin levels were measured using a mouse leptin ELISA kit, according to the manufacturer’s instructions (Abcam, ab100718).

### Cell lines and culture conditions.

GBC cell lines RCB-1130 and SNU-308 were gifts from Chien-Feng Li at the Department of Medical Research, Chi Mei Medical Center. RCB-1130 cells were maintained in Dulbecco’s modified Eagle’s medium supplemented with 10% fetal bovine serum, 100 units/ml penicillin, and 100 mg/ml streptomycin. SNU-308 cells were maintained in RPMI 1640 medium supplemented with 10% fetal bovine serum, 100 units/ml penicillin, and 100 mg/ml streptomycin.

### Preparation of adipocyte CM.

The ADSC isolation protocols were established by Patricia Zuk and Marc Hedrick at UCLA (31). Culture medium was supplemented with 0.5 mM isobutyl-methylxanthine (MilliporeSigma), 1 μM dexamethasone (MilliporeSigma), 10 μg/ml insulin (MilliporeSigma), and 1 μM indomethacin (MilliporeSigma). Lipid droplets were observed in differentiated mature adipocytes, but not in ADSCs, as demonstrated by phase images and Oil Red O staining for the accumulation of lipid and fat. Next, the media were replaced with serum-free DMEM medium on day 2. After a 24-hour culture period, the media were collected and replaced with fresh serum-free medium. Then, CM were collected every 24 hours for incubation for 3 days. The CM were centrifuged for 15 minutes at 800*g* to eliminate detached cells and cell debris and frozen at 20°C before use.

### Cell viability assay.

Cell survival was measured using diluted 3-(4,5-cimethylthiazol-2-yl)-2,5-diphenyl tetrazolium bromide (MTT) (MilliporeSigma) reagent for 4 hours. The samples were then measured spectrophotometrically at 595 nm using an ELISA plate reader. Experimental cells were treated with gemcitabine (10–1000 nM) (MilliporeSigma) for 24 hours. For combination treatment, cells were treated with gemcitabine or adipocyte CM or leptin 100 ng/mL (R&D Systems) and MCL1 inhibitor (MIM1) (TOCRIS) for 24 hours. The percentage cell viability and death for each treatment were calculated by normalizing to the untreated control group.

### LDH assay.

RCB-1130 cells were seeded (5 × 10^3^ cells/well) in 96-well plates. Cells were treated with 100 nM gemcitabine or 100 ng/mL leptin combined with gemcitabine for 24 hours. Then, the experimental cells were incubated with reconstitute substrate mix at 37°C for 30 minutes according to the manufacturer’s instructions (J2380, Promega). The samples were then measure spectrophotometrically at 490 nm by an ELISA reader.

### TUNEL staining assay of apoptosis.

Following the applied treatment, the TUNEL In Situ Cell Death Detection Kit (Roche) was utilized to evaluate cell apoptosis. The cell apoptosis ratio was calculated using the TUNEL percentage (TUNEL/DAPI × 100%).

### Caspase-3 activity assay.

The activity of caspase-3 was assayed by the activity assay kit (ab39383, Abcam) according to the manufacturer’s protocols. Briefly, the assay was based on the detection of cleavage of the fluorogenic substrate DEVD-AFC (7-amino-4-trifluoromethyl coumarin). DEVD-AFC emitted blue light (maximum = 400 nm); upon cleavage of the substrate by caspase-3, free AFC emitted a yellow-green fluorescence (excitation/emission = 400/505 nm), which could be quantified using a microplate reader (BioTec).

### SubG1 analysis.

RCB-1130 cells were seeded (1 *×* 10^5^/well) in 6-well plate. Cells were preinfected with lentiviruses encoding shβ-galactosidase (sh-control) or shmcl-1 or shmfn-1 for 48 hours to knockdown gene expression. The short interfering RNA sequences targeted to void mcl-1 and mfn-1 were subcloned into the lentiviral expression vector, PLKO. The sequences of short interfering RNA are shown as follows: shβ-galactosidase, 5′-AGTTCAGTTACGATATCATGTCTCGAGACATTCGCGAGTAACTGAACTTTTTTG-3′; shmcl-1, 5′-CCGGGCTGTGTTAAACCTCAGAGTTCTCGAGAACTCTGAGGTTTAACACAGCTTTTT-3′; and shmfn-1, 5′-CCGGTAGTGGGATTGGCCATATAACCTCGAGGTTATATGGCCAATCCCACTATTTTTG-3′. Then, cells were treated with 100 nM gemcitabine combined with 100 ng/mL leptin for 24 hours. Cells were harvested, and then cells were washed with 1X PBS and fixed with 70% ethanol at –20°C and then stained with 20 μg/ml propidium iodide, 20 μg/ml RNase A, and 0.1% Triton X-100. Samples were analyzed using flow cytometry (CellLab Quanta SC, Beckman Coulter).

### Lentiviral knockdown assay.

The viruses were produced from Phoenix cells by the cotransfection of various shRNA expression vectors in combination with pMD2.G and psPAX2. The lentiviral knockdown expression vectors were obtained from the National RNAi Core Facility located at the Genomic Research Center of the Institute of Molecular Biology, Academia Sinica, Taipei, Taiwan. After determining the viral infection efficiency, lentiviruses that contained sh-galactosidase (shLacZ) or shCEBPD were used to infect the SNU-308 and RCB-1130 cells for 72 hours. In all lentiviral experiments, the medium containing the uninfected viruses was removed before conducting further assays. The shRNA sequences in the lentiviral expression vectors were as follows: shβ-galactosidase, 5′-CCGGTGTTCGCATTATCCGAACCATCTCGAGATGGTTCGGATAATGCGAACATTTTTG-3′, and shCEBPD, 5′-CCGGGCCGACCTCTTCAACAGCAATCTCGAGATTGCTGTTGAAGAGGTCGGCTTTTT-3′.

### Western blot analysis.

Experimental cells were harvested and lysed with modified RIPA buffer (50 mM Tris-HCl [pH 7.4], 150 mM sodium chloride, 1 mM EDTA, 1% NP40, 0.25% sodium deoxycholate, 1 mM dithiothreitol, 10 mM NaF, 1 mM PMSF, 1 μg/mL aprotinin and 1 μg/mL leupeptin). Lysates were resolved on SDS-containing 10% polyacrylamide gel, transferred to PVDF membranes, and probed with specific antibodies as follows: α-tubulin (MilliporeSigma, T9026), CEBPD (Santa Cruz Biotechnology, sc-365546), phospho STAT3 (Y705) (Abcam, ab76315), STAT3 (Abcam, ab68153), MCL1 (Abcam, ab32087), BCL-2 (GeneTex, GTX61005), and BCL-XL (GeneTex, GTX105661). Enhanced chemiluminescence was purchased from Amersham Life Sciences Inc.

### Quantitative real-time PCR.

The isolated RNAs were subjected to RT reactions using SuperScript III (Invitrogen) for cDNA synthesis. The resultant cDNA was mixed with KAPA SYBR FAST qPCR Master Mix (Life Technologies Corporation and Kapa Biosystems Inc.) and appropriate primers, and quantitative PCR was performed in a Thermocycler C1000. The following specific primers were used for the quantitative real-time PCR analysis: CEBPD, 5′-ACTCAGCAACGACCCATACC-3′ and 5′-CGCTCCTATGTCCCAAGAAA-3′, and MCL1, 5′-AGAAAGCTGCATCGAACCAT-3′ and 5′-CCAGCTCCTACTCCAGCAAC-3′.

### Plasmid transfection and reporter gene assay.

RCB-1130 cells were seeded at an optimal density 12 hours before transfection in 2 ml fresh culture medium in a 6-well plastic plate. The cells were then transfected with plasmids using TurboFect (Thermo Fisher Scientific) according to the manufacturer’s instructions. The total amount of DNA for each experiment was matched to their individual backbone vectors. Opti-MEM was changed to CM, and the cells were incubated for 16 hours. The PCDNA3/CEBPD (CD) plasmid and MCL1 reporter –266/+50 were transfected. After transfection, the luciferase activities of the cell lysates were measured using the luciferase assay system as per the manufacturer’s instructions (Promega).

### Mitochondrial morphology analysis.

RCB-1130 cells were cultured on coverslips in 35 mm dishes in 2 ml DMEM supplemented with 10% FCS at 37°C overnight under an atmosphere of 10% CO_2_ in air. The cells were incubated for 24 hours. When mitochondria were stained, 100 nM MitoTracker (Molecular Probes) was added to the medium and incubated for 20 minutes before fixation. The cells cultured on coverslips were fixed with 2 ml 50% acetone or 50% methanol for 5 minutes at room temperature. The coverslips were washed with 2 ml PBS 3 times. Fluorescent images were taken and analyzed by a confocal laser microscope (Radiance 2000, Bio-Rad Laboratories).

### Mitochondrial stress analysis.

To analyze the mitochondrial stress in GBC cells, we used the Seahorse XFp analyzer (Seahorse Bioscience). Cells were plated on 24 wells overnight in Seahorse XFp miniplates. On the day of assay, pharmaceutical compounds, including oligomycin (1 μM), FCCP (0.2 μM), and antimycin A (1 μM), within the Seahorse cell energy phenotype test kit were reconstituted and used to make to a stressor mix at optimized concentration. The OCR was then recorded and analyzed by Wave software (Seahorse). At least 3 biological independent experiments were conducted in triplicates.

### MMP.

MMP was measured using JC-1 dye (Abcam, ab113850), according to manufacturer’s instructions. Briefly, cells were incubated with 5 μM JC-1 for 20 minutes, then cells were washed twice with the completed medium and fluorescence was detected by flow cytometry (BD Biosciences).

### Statistics.

The statistical significance of the differences between the mean values was estimated using the SigmaPlot software package, with the independent 2-tailed Student’s *t* test and 1-way ANOVA followed by Tukey’s multiple comparisons for unequal variances. The χ^2^ test or Fisher’s exact probability test was used to compare clinicopathological features of the patients with p-STAT3, CEBPD, and MCL1 expression. Cox proportional regression analysis with adjusted confounding factors was used for survival analysis. Correlation analysis of p-STAT3, CEBPD, and MCL1 expression was evaluated using Pearson’s correlation analysis. The data are expressed as the mean ± SEM. *P* values of less than 0.05 were considered statistically significant.

### Study approval.

The patients with GBC provided consent for the use of tumor tissue for clinical research, and the Shanghai Jiao Tong University Xinhua Hospital Ethical Committee approved the research protocol. The human ADSCs were isolated from obese but metabolically healthy donor subcutaneous tissues via liposuction with informed consents to protect patient privacy and rights, as approved by the Institutional Review Board of the National Cheng Kung University Hospital. All animal studies were carried out using procedures approved by the Institutional Animal Care and Use Committee of National Cheng Kung University (approval 103209). The animal experiments were also performed to conform to the NIH guidelines and the *Guide for the Care and Use of Laboratory Animals* (National Academies Press, 2011).

## Author contributions

WJW, HY Lai, and JMW conceived the study. WJW, HY Lai, FZ, WJS, PYC, HY Liang, and YBL contributed to the methodology. WJW, HY Lai, FZ, WJS, and PYC contributed to the investigation. WJW, HY Lai, FZ, WJS, PYC, and HY Liang provided formal analysis. WJW, HY Lai, FZ, YBL, and JMW provided resources. WJW and HY Lai contributed to the validation. WJW, HY Lai, and PYC contributed to data visualization. WJW and HY Lai wrote the original draft of the manuscript. WJW, HY Lai, and JMW reviewed and edited the manuscript. WJW and JMW acquired funding. JMW supervised the study.

## Supplementary Material

Supplemental data

## Figures and Tables

**Table 1:**
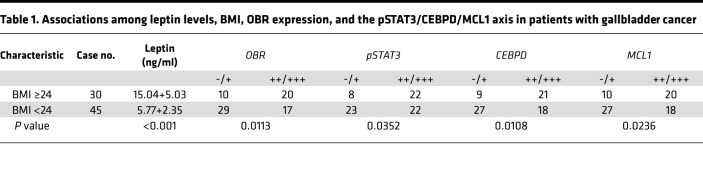
Associations among leptin levels, BMI, OBR expression, and the pSTAT3/CEBPD/MCL1 axis in patients with gallbladder cancer

**Table 2:**
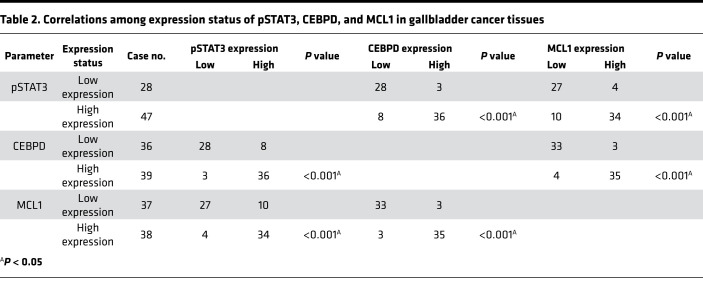
Correlations among expression status of pSTAT3, CEBPD, and MCL1 in gallbladder cancer tissues

## Version 1 06/22/2021

In-Press Preview

## Version 2 08/09/2021

Electronic publication
