# Supplementary material for: MCL1 participates in leptin-promoted mitochondrial fusion and contributes to drug resistance in gallbladder cancer
Source: JCI Insight. 2021 Aug 9;6(15):e135438. doi: 10.1172/jci.insight.135438 (PMC8410075; doi:10.1172/jci.insight.135438)
Supplement: Supplemental data [file jciinsight-6-135438-s286.pdf]

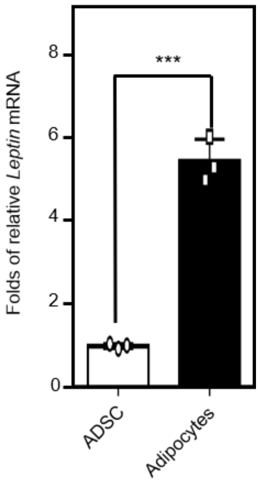

Supplementary Figure 1. *Leptin* transcripts are abundant in human adipocytes.

qPCR assay was conducted with total RNAs harvested from mesenchymal stem cells and adipocytes. n=3, 2-tailed Student’s t-test. ***, significant difference (P < 0.001).

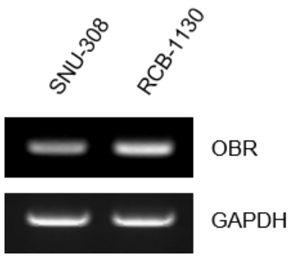

Supplementary Figure 2. OBR is expressed in gallbladder cancer cells.

RT-PCR assay was conducted with total RNA harvested from SNU-308 and RCB-1130 gallbladder cancer cells. (n=3)

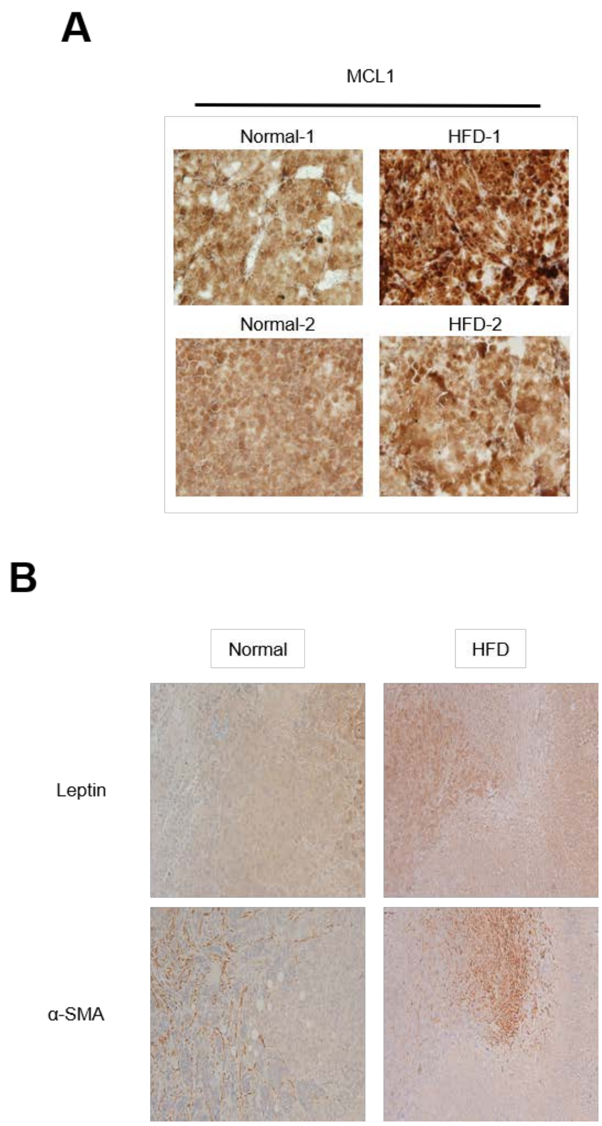

Supplementary Figure 3. MCL1 expression is induced in the tumor xenografts of HFD-treated mice.

A, MCL-1 expression is higher in RCB-1130 xenograft HFD-treated obese NOD/SCID mice. After injecting gallbladder cancer RCB-1130 cells into normal and HFD-treated obese mice for 4 weeks, the xenograft mice were sacrificed to extract tumor xenografts. MCL-1 protein expression was then examined by immunohistochemistry. (n=4)

B, The expressions of leptin (an adipocyte marker) and α-SMA (a fibroblast marker) are marginally induced in the HFD-treated tumor xenografts. The leptin and α-SMA protein expressions were then examined by immunohistochemistry. (n=4)

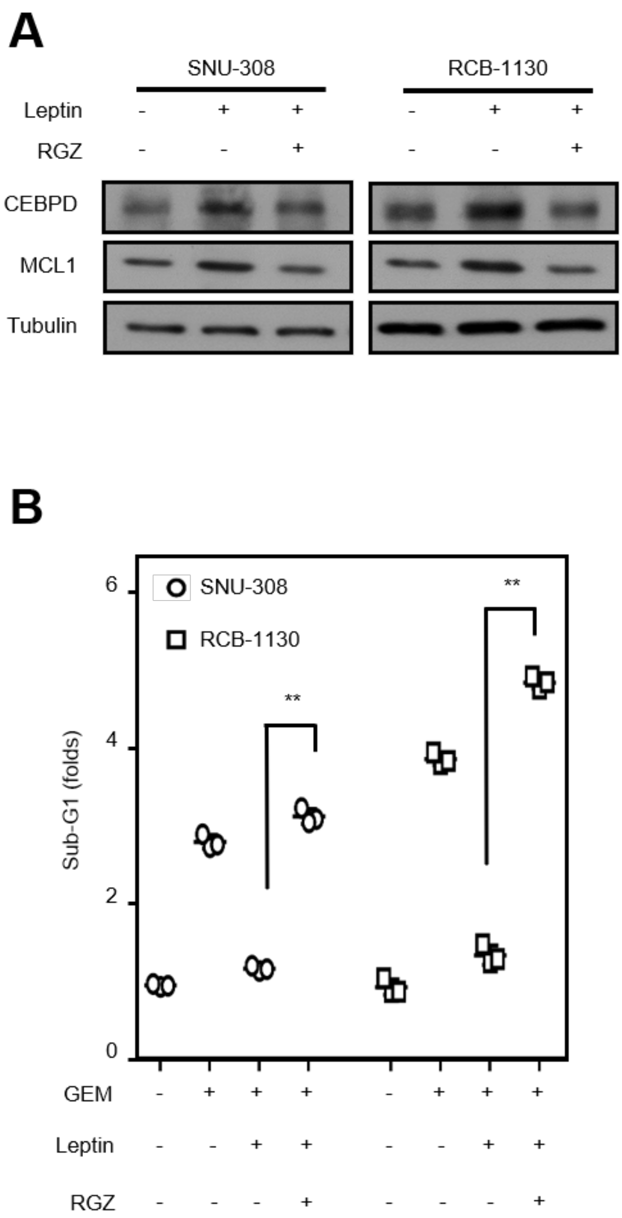

Supplementary Figure 4. PPARγ agonist rosiglitazone suppresses leptin-induced CEBPD and MCL1 expression and enhances GBC cells sensitivity to gemcitabine.

A, PPARγ agonist rosiglitazone (RGZ) inhibits leptin-induced pathway in GBC cells. A Western blot was conducted using lysates of leptin-treated alone or in combination treatment with RGZ in SNU-308 and RCB-1130 cells and detected with indicated specific CEBPD, MCL1 and α-tubulin antibodies. (n=3)

B, RGZ enhances GBC cells sensitivity to GEM. SNU-308 and RCB-1130 cells were treated with GEM alone or in combination treatment with leptin or RGZ. n=3, 2-tailed Student’s t-test.

Supplementary Table 1. Correlation of pSTAT3, CEBPD and MCL1 expression with clinicopathological features in gallbladder cancer specimens.

characteristic	Case No.	*pSTAT3*			*CEBPD*			*MCL1*		
		-/+	++/+++	*p*	-/+	++/+++	*p*	-/+	++/+++	*p*
**Age**										
<63	33	13	20		15	18		16	17	
≥63	42	18	24	0.762	21	21	0.696	21	21	0.896
**Gender**										
Male	21	11	10		13	8		14	7	
Female	54	20	34	0.226	23	31	0.133	23	31	0.061
**Tumor differentiation**										
Well/moderate	40	19	21		23	17		24	16	
Poor	35	12	23	0.246	13	22	0.078	13	22	0.048
**TNM stage (AJCC)**										
I/II	29	21	8		27	2		24	5	
III/IV	46	10	36	< 0.0001	9	37	< 0.0001	13	33	< 0.0001
**Metastasis**										
Present	40	10	30		9	31		12	28	
Absent	35	21	14	0.002	27	8	< 0.0001	25	10	3E-04
**Microvascular and Neural invasion**										
Present	42	17	25		16	26		18	24	
Absent	33	14	19	0.865	20	13	0.053	19	14	0.206
